# Non-Invasive Prediction of Survival Time of Midline Glioma Patients Using Machine Learning on Multiparametric MRI Radiomics Features

**DOI:** 10.3389/fneur.2022.866274

**Published:** 2022-05-02

**Authors:** Da-Biao Deng, Yu-Ting Liao, Jiang-Fen Zhou, Li-Na Cheng, Peng He, Sheng-Nan Wu, Wen-Sheng Wang, Quan Zhou

**Affiliations:** ^1^Department of Radiology, Third Affiliated Hospital of Southern Medical University (Academy of Orthopedics Guangdong), Guangzhou, China; ^2^Imaging Department of Guangdong 999 Brain Hospital, Guangzhou, China; ^3^GE Healthcare, Guangzhou, China; ^4^Department of Neuro-Oncology of Guangdong 999 Brain Hospital, Guangzhou, China

**Keywords:** machine learning, radiomics, MRI, midline glioma, overall survival

## Abstract

**Objectives:**

To explore the feasibility of predicting overall survival (OS) of patients with midline glioma using multi-parameter magnetic resonance imaging (MRI) features.

**Methods:**

Data of 84 patients with midline gliomas were retrospectively collected, including 40 patients with OS > 12 months (28 cases were adults, 14 cases were H3 K27M-mutation) and 44 patients with OS < 12 months (29 cases were adults, 31 cases were H3 K27M-mutation). Features were extracted from the largest slice of tumors, which were manually segmented on T2-weighted (T2w), T2 fluid-attenuated inversion recovery (T2 FLAIR), and contrast-enhanced T1-weighted (T1c) images. Data were randomly divided into training (70%) and test cohorts (30%) and normalized and standardized using Z-scores. Feature dimensionality reduction was performed using the variance method and maximum relevance and minimum redundancy (mRMR) algorithm. We used the logistic regression algorithm to construct three models for T2w, T2 FLAIR, and T1c images as well as one combined model. The test cohort was used to evaluate the models, and receiver operating characteristic (ROC) curves, areas under the curve (AUCs), sensitivity, specificity, and accuracy were calculated. The nomogram of the combined model was built and evaluated using a calibration curve. Decision curve analysis (DCA) was used to evaluate the clinical application value of the four models.

**Results:**

A total of 1,316 features were extracted from T2w, T2 FLAIR, and T1c images, respectively. And then the best non-redundant features were selected from the extracted features using the variance method and mRMR. Finally, five features were extracted each from T2w, T2 FLAIR, and T1c images, and 12 features were extracted for the combined model. Four models were established using the optimal features. In the test cohort, the combined model performed the best out of all models. The AUCs of the T2w, T2 FLAIR, T1c, and combined models were 0.73, 0.78, 0.74, and 0.87, respectively, and accuracies were 0.72, 0.76, 0.72, and 0.84, respectively. The ROC curves and DCA showed that the combined model had the highest efficiency and most favorable clinical benefits.

**Conclusion:**

The combined radiomics model based on multi-parameter MRI features provided a reliable non-invasive method for the prognostic prediction of midline gliomas.

## Introduction

Diffuse midline gliomas (DMGs) with an H3 K27M mutation are a group of tumors newly defined in 2016 and amended to DMGs with an H3 K27-altered in 2021 (1, 2). They occur commonly in the brainstem of children, although they are not uncommon in adults. The median survival time of patients with a DMG with H3 K27M mutation is shorter than that of patients without H3 K27M mutation (3). Furthermore, H3 K27M mutation not only have prognostic implications but may also represent a potential new immunotherapeutic target (4). However, because of the high risk of operating on the midline area, most patients only undergo radiotherapy or chemotherapy (5, 6). Without information regarding H3 K27-altered, predicting the survival of patients with midline gliomas is challenging. Therefore, it is of significant clinical value to develop a non-invasive method to evaluate the survival of patients with midline gliomas.

In clinical practice, magnetic resonance imaging (MRI) is one of the most valuable methods for evaluating the survival of patients with midline gliomas before and during treatment. MRI findings of tumors often help clinicians adjust treatment strategies. Furthermore, the World Health Organization's classification of tumors of the central nervous system began integrating molecular and genetic profiling in 2016, with gradual improvements being made continuously. However, the relationship between macroscopic MRI findings and molecular subtyping of tumors remains uncertain. H3 K27M mutant patients has shorter survival time than those of wild type, and several studies have shown no significant correlation between H3 K27M mutant- and wild-type DMGs in terms of tumor necrosis, patterns of enhancement, edema, infiltrative features, or diffusion characteristics (3, 7, 8). This suggests that macroscopic MRI findings, such as tumor location, size, signal intensity, contrast enhancement, and advanced MRI techniques, such as diffusion-weighted imaging, do not currently meet the clinical needs of evaluation of DMG survival. On the other hand, according to the 2021 WHO classification of tumors of the central nervous system, DMGs, H3 K27-altered include H3 wild type with enhancer of zeste homolog inhibitory protein (EZHIP) overexpression and epidermal growth factor receptor (EGFR) mutation in addition to H3 K27M mutation (2). Therefore, the prediction of H3 K27M mutation alone in the past cannot accurately evaluate the prognosis of patients with DMG. Thus, there is an urgent need for better and more advanced tools to explore biological information hidden in traditional MR images, which would have substantial clinical implications, such as avoiding inappropriate treatments.

One such tool is radiomics, which involves the non-invasive extraction of a large amount of quantitative information from medical images that cannot be perceived by human vision. To date, radiomics has been demonstrated to be valuable in predicting the molecular genetic characteristics, tumor grading, differential diagnosis, and prognostic evaluation of gliomas (6, 9–14). Numerous studies have accurately predicted the survival of gliomas using radiomics. Prasanna et al. (15) found that radiomic features from the peritumoral brain parenchyma on routine pre-operative MRI can predict long- vs. short-term survival in glioblastomas. Senders et al. (16) have trained fifteen statistical and machine learning algorithms based on 13 demographic, socioeconomic, clinical, and radiographic features to predict overall survival, 1-year survival status of glioblastomas, and found the accelerated failure time model demonstrated superior performance compared to Cox proportional hazards regression and other machine learning algorithms. Therefore, we investigated the feasibility of predicting the overall survival (OS) of patients with midline gliomas using machine learning to offer an alternative method to non-invasively predict prognoses of midline glioma patients.

## Materials and Methods

### Patients

This study was approved by the hospital ethics committee, and the requirement for obtaining informed consent was waived. We retrospectively collected data of patients with a midline glioma treated at Guangdong 999 Brain Hospital between January 2017 and October 2020. The inclusion criteria were: (1) glioma located in the midline area (e.g., vermis, brainstem, thalamus, corpus callosum, sellar region, and basal ganglia); (2) patients had undergone surgical resection or biopsy, and the pathological diagnosis was certain; and (3) patients underwent T2-weighted (T2w), T2 fluid-attenuated inversion recovery (T2 FLAIR), and contrast-enhanced T1-weighted (T1c) imaging at our hospital before the operation. Exclusion criteria were: (1) missing T2w, T2 FLAIR, or T1c images; (2) uncertain pathological diagnosis; and (3) poor image quality. Poor image quality is defined as obvious motion artifacts; the vascular pulsation artifact in the posterior fossa was obvious after enhancement; and (4) patients who had received related preoperative treatments, such as radiotherapy or chemotherapy; (5) patients who had been followed up for < 12 months.

**Figure 1:**
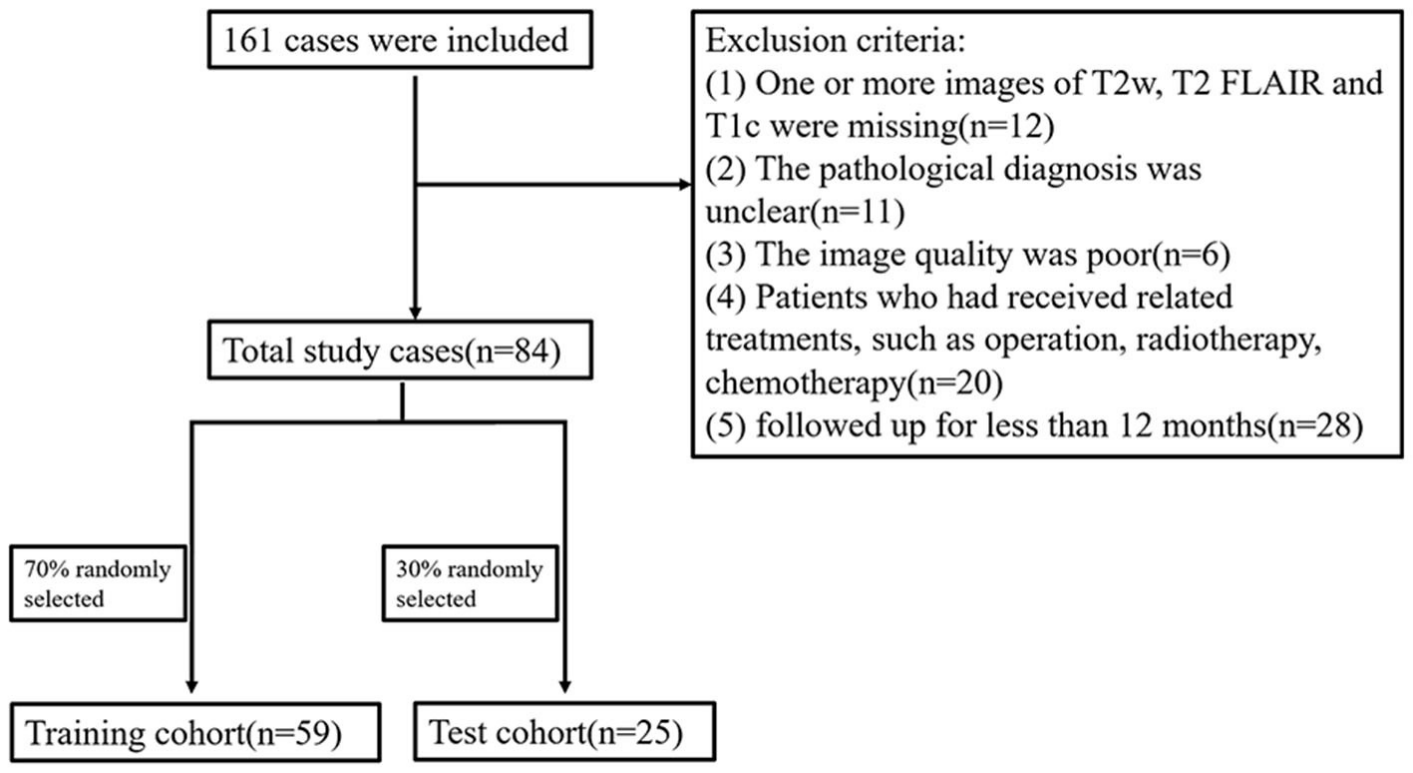
Flowchart of patient inclusion and patient groupings.

Initially, 161 cases were included, of whom 77 were excluded; 20 had received treatment before surgery, 11 had uncertain pathology, 12 had incomplete imaging data, 6 had poor image quality, and 28 were followed up for < 12 months. Finally, a total of 84 cases were included. The OS of 40 cases was >12 months (long-term group) and the OS of 44 cases was <12 months (short-term group) (Figure 1).

### T1w, T2w, T2 FLAIR, and T1c Images Acquisition

All patients were scanned using a GE HDxt 3.0T MR (General Electric, Milwaukee, WI, USA) or Philips Intera 1.5T MR (Royal Philips Electronics, Amsterdam, Netherlands) scanner. Imaging sequences included T1w, T2w, T2 FLAIR, and T1c sequences. The 3.0T MRI scan parameters were: repetition time (TR) 1,900 ms, echo time (TE) 24 ms, and inversion time (TI) 780 ms for T1w imaging; TR 4,480 ms and TE 120 ms for T2w imaging; TR 9,480 ms, TE 120 ms, and TI 2,300 ms for T2 FLAIR imaging. The T1w, T2w, and T2 FLAIR imaging sequences on the 3.0T scanner used the following parameters: field of view (FOV): 240 × 240 mm, matrix: 256 × 256, slice thickness: 5.5 mm, slice gap: 1.0 mm, number of excitations (NEX): 1. The 1.5T MRI scan parameters were: TR 488 ms, TE 15 ms, and matrix: 152 × 121 for T1w imaging; TR 3,980 ms, TE 110 ms, and matrix: 230 × 130 for T2w imaging; TR 6,000 ms, TE 120 ms, TI 2,000 ms, and matrix: 192 × 115 for T2 FLAIR imaging. The T1w, T2w, and T2 FLAIR imaging sequences on the 1.5T scanner used the following parameters: FOV: 230 × 182 mm, slice thickness: 5.5 mm, slice gap: 1.0 mm, NEX: 1. T1c imaging on the 1.5T and 3.0T MRI scanners was performed after the administration of Gadolinium-Diethylenetriaminepentaacetic Acid (Gd-DTPA) (Kangchen Company, Guangzhou, China) or Gd diamine (GE Pharmaceuticals, USA) at a dose of 0.1 mmol/kg body weight.

### Tumor Segmentation

**Figure 2:**
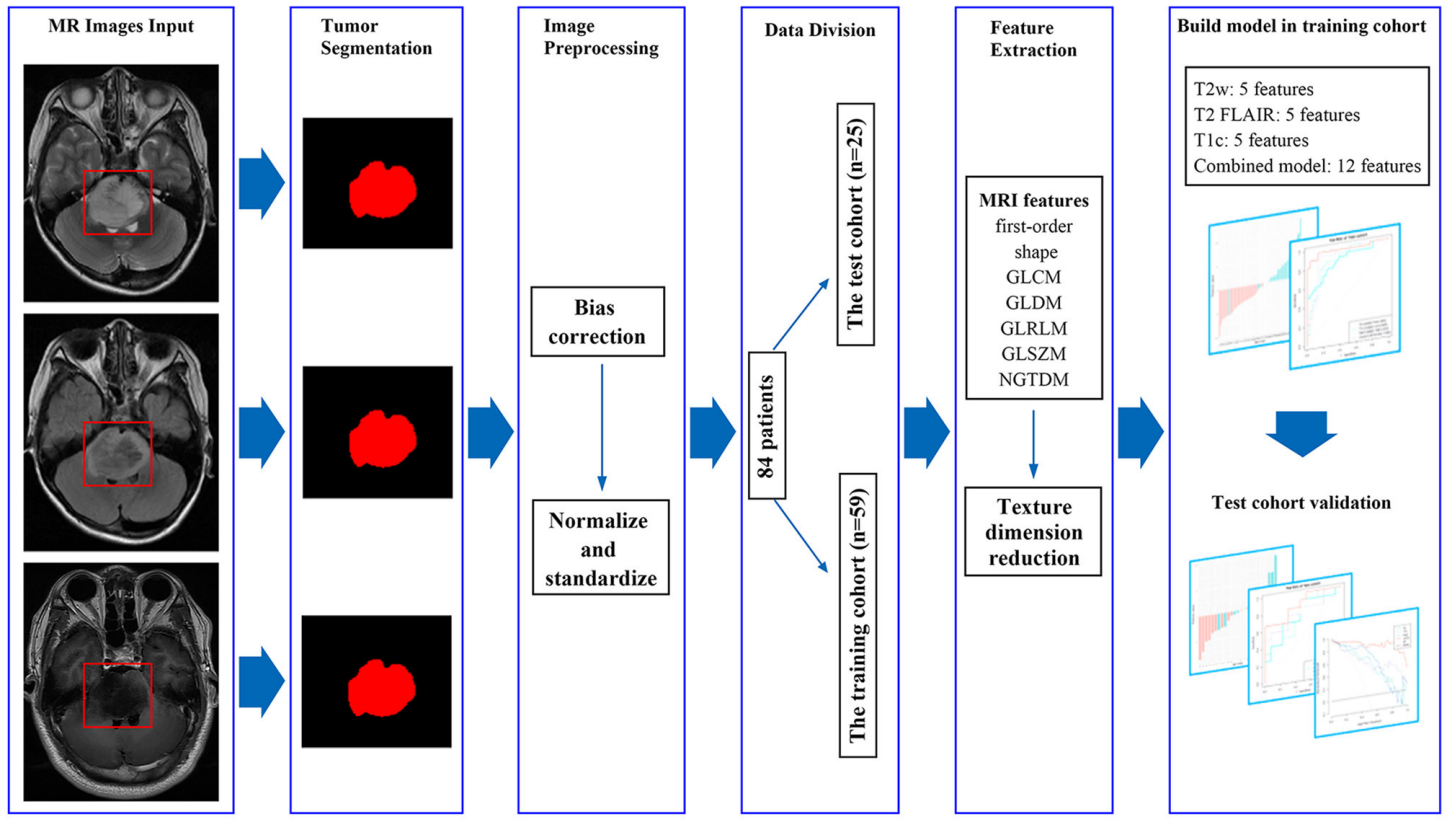
MRI feature extraction and machine learning flow chart. By manually segmenting the tumors, we extracted 1,316 imaging features from masked preoperative T2w, T2 FLAIR, and T1c images. The variance method and maximum relevance and minimum redundancy were used to select the best features, and the four models were established. The test cohort was then used to verify the model.

All images were saved as Digital Imaging and Communications in Medicine (DICOM) and imported into the ITK-SNAP software (version 3.8.0, http://www.itksnap.org) for segmentation. Some studies have shown that one slice (2D) with the largest cross-section of the tumor and the entire tumor volume (3D) for segmentation has comparable diagnostic performance, and there are also some studies that use one slice for tumor segmentation (17–20). So, in this study, the regions of interest (ROIs) were manually segmented (Figure 2) on the axial slice with the largest cross-section of the tumors on T2w, T2 FLAIR, and T1c images by two authors (S Wu and P He, with 4 and 6 years of diagnostic experience in neuroradiology, respectively) who were blinded to histological results.

The final ROIs were calculated using the overlapping segmentations generated by the two authors. If the overlap rate was <90%, the ROI was defined by L Cheng, who had >10 years of diagnostic experience in neuroradiology (16, 21). The ROIs included cystic and necrotic components which may reflect tumor heterogeneity.

### Imaging Feature Extraction

A total of 1,316 texture parameters were extracted from T2w, T2 FLAIR, and T1c images using the open-source package Pyradiomics (version 3.0.1, https://github.com/Radiomics/pyradiomics) on Python (version 3.5.6, https://www.python.org) and included first-order statistical features, shape features, gray level co-occurrence matrix, gray level dependence matrix, gray level run length matrix, gray level size zone matrix, and neighboring gray tone dependence matrix (22). The median and block methods were used to replace missing values and outliers.

### Data Cohort Division and Preprocessing

Data were randomly divided into training (70%; 59 patients including the long-term group 31 patients and the long-term group 28 patients) and test (30%; 25 patients including the long-term group13 patients and the long-term group 12 patients) cohorts. All features extracted from the ROIs were normalized using Z-scores.

### Feature Dimensionality Reduction and Selection of Optimal Features

The variance method was used to calculate the variance of features extracted from imaging. When the variance of the feature was 0, the feature was removed. The maximum relevance and minimum redundancy (mRMR) method was used to select the best non-redundant features from the extracted features.

### Radiomics Feature Model and Evaluation

Logistic regression was used to establish the model. According to the best features of the T2w, T2 FLAIR, and T1c images, three models for T2w, T2 FLAIR, and T1c images and one combined model (T2w + T2 FLAIR + T1c) were constructed. Receiver operating characteristic (ROC) curve analysis and decision curve analysis (DCA) were used to assess the performance of the models (Figure 2).

### Statistical Analysis

All statistical analyses were performed using R (version 3.5.1, http://www.R-project.org) and Python (version 3.5.6, https://www.python.org/). Chi-squared or Fisher's exact tests were used for nominal variables. Kruskal–Wallis H tests were used for ordinal variables. Student's t-tests were used for continuous variables. The variance method and mRMR were used for feature dimensionality reduction. Logistic regression was used to establish the model and construct a nomogram. ROC curve analysis was used to assess the established models according to accuracy (AC), area under the curve (AUC), sensitivity, and specificity values. DCA was used to evaluate the application value of the models. A *p* < 0.05 was considered statistically significant.

## Results

### Patient Characteristics

**Figure 3:**
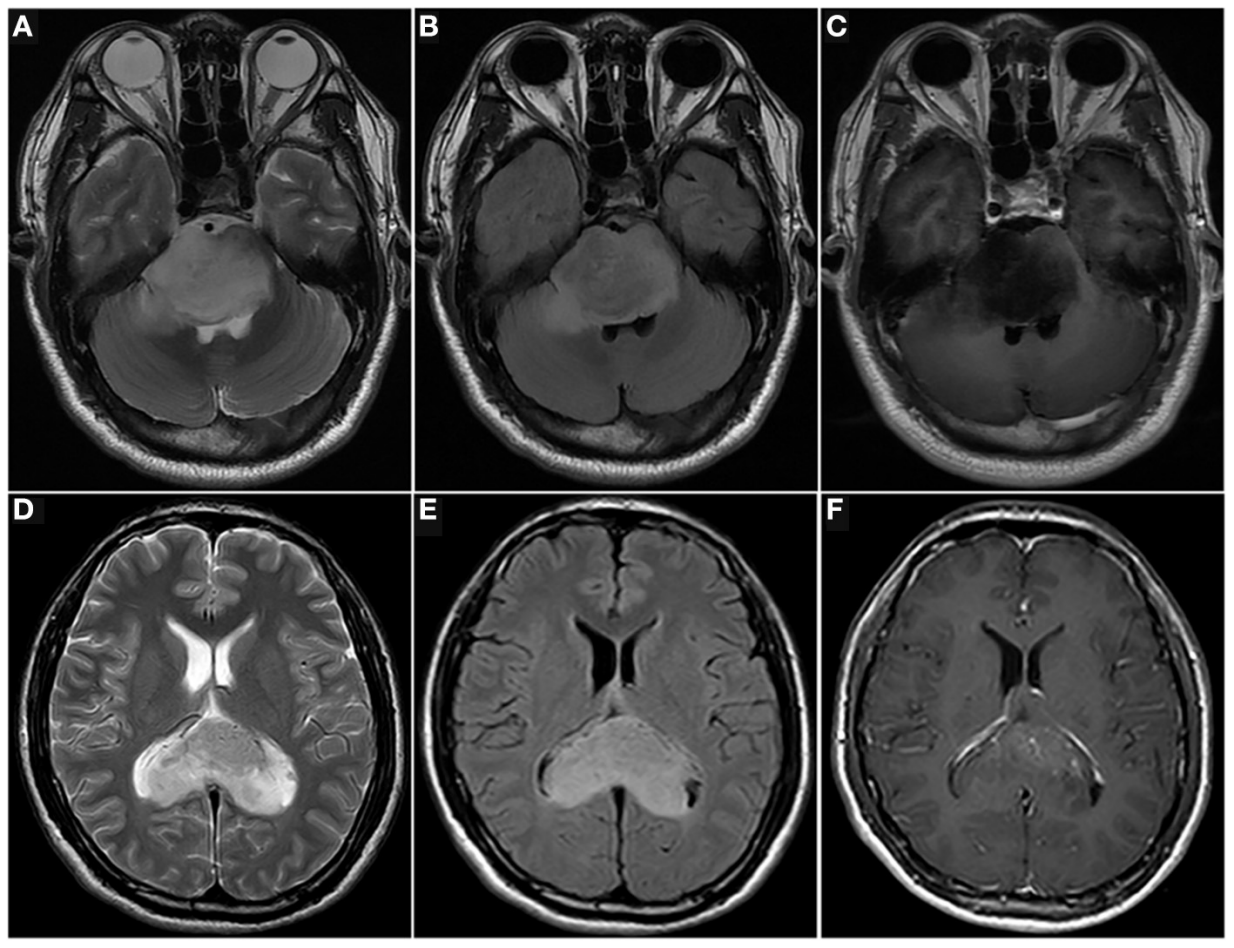
Images of patients with midline gliomas. T2w **(A)**, T2 FLAIR **(B)**, and T1c **(C)** images of a 34-year-old man with a pontine diffuse midline glioma with a H3 K27M mutation (WHO 4), and histopathology of diffuse astrocytoma. He underwent a biopsy and was treated with radiotherapy and chemotherapy. The overall survival of the patient was 4.4 months. T2w **(D)**, T2 FLAIR **(E)**, and T1c **(F)** images of a 33-year-old man with corpus callosum diffuse astrocytoma (WHO 2). He underwent a biopsy and was treated with radiotherapy and chemotherapy. The overall survival of the patient was 39 months (up to October 2020).

A total of 84 patients with a midline glioma were enrolled in the study (62 men and 22 women; mean age 27.73 ± 16.36 years; range 1–63 years). The clinical characteristics of the long-term and short-term groups are shown in Table 1, Figure 3. There was a significant difference in radiotherapy between the long-term group and the short-term group (*p* = 0.002). The patients with H3 K27M mutation in short-term group outnumbered those in long-term group significantly (*p* = 0.001). There was also a significant difference in OS between the short-term and long-term groups (5.16 ± 5.38 months vs. 25.34 ± 9.46 months, *p* < 0.001). The clinical characteristics of the training and test cohorts are shown in Table 2. The patients who underwent radiotherapy in the training outnumbered those in the test cohort significantly (*p* = 0.0028).

**Table 1:** Clinical characteristics of 84 patients in the short-term and long-term groups.

**Variable**		**Cases**	**Short-term group (*n* = 44)**	**Long-term group (*n* = 40)**	**Statistical value**	***p*-value**
Men		62	35 (79.55%)	27 (67.50%)	1.573	0.21
Women		22	9 (20.45%)	13 (32.50%)		
Age		84	27.25 ± 17.51	28.25 ± 15.19	−0.278	0.781
	Age (<18 years)	27	15 (34.09%)	12 (30.0%)	0.161	0.688
	Age (≥18 years)	57	29 (65.91%)	28 (70.0%)		
Tumor classification						
	WHO^*^ 1	14	2 (4.55%)	12 (30.0 %)	11.868	0.001
	WHO 2	21	9 (20.45%)	12 (30.0 %)		
	WHO 3	19	12 (27.27 %)	7 (17.50 %)		
	WHO 4	30	21 (47.73 %)	9 (22.50 %)		
H3 K27M-mutation		45	31 (70.45%)	14 (35.00%)	10.589	0.001
H3 K27M wild-type		39	13 (29.55%)	26 (65.00%)		
Tumor location						
	Sellar region	4	2 (4.55%)	2 (5.0%)	0.029	0.865
	Basal ganglia	4	2 (4.55%)	2 (5.0%)		
	Brainstem	32	15 (34.09%)	17 (42.50%)		
	Corpus callosum	8	5 (11.36%)	3 (7.50%)		
	Thalamus	31	20 (45.45%)	11 (27.50%)		
	Cerebellar vermis	5	0	5 (12.50%)		
Operation						
	Biopsy	21	14 (31.82%)	7 (17.50%)	3.377	0.066
	Partial resection	31	16 (36.36%)	15 (37.50%)		
	Subtotal resection	23	12 (27.27%)	11 (27.50%)		
	Total resection	9	2 (4.55%)	7 (17.50%)		
Treatment						
	Radiotherapy	55	22 (50.0%)	33 (82.50%)	9.79	0.002
	Without radiotherapy	29	22 (50.0%)	7 (17.50%)		
	Chemotherapy	38	13 (29.55%)	25(62.50%)	1.285	0.257
	Chemotherapy + targeted therapy	11	8 (18.18%)	3 (7.50%)		
	Without chemotherapy	35	23 (52.27%)	12 (30.0%)		
OS^**^		84	5.16 ± 5.38	25.34 ± 9.46	−11.865	<0.001

* *World Health Organization*.

** *Overall survival*.

**Table 2:** Clinical characteristics of 84 patients in the training and test cohorts.

**Variable**		**Cases**	**Training cohort**	**Test cohort**	**Statistical value**	***p*-value**
Men		62	45 (76.27%)	17 (68.0%)	0.621	0.431
Women		22	14 (23.73%)	8 (32.0%)		
Age		84	29.15 ± 17.07	24.36 ± 14.29	−1.232	0.222
	Age (<18 years)	27	18 (30.51%)	9 (36.0%)	0.243	0.622
	Age (≥ 18 years)	57	41 (69.49%)	16 (64.0%)		
Tumor classification						
	WHO^*^ 1	14	8 (13.56%)	6 (24.0%)	0.146	0.703
	WHO 2	21	15 (25.42%)	6 (24.0%)		
	WHO 3	19	16 (27.12%)	3 (12.0%)		
	WHO 4	30	20 (33.90%)	10 (40.0%)		
H3 K27M-mutation		45	30 (50.85%)	15 (60.00%)	0.591	0.442
H3 K27M wild-type		39	29 (49.15%)	10 (40.00%)		
Tumor location						
	Sellar region	4	4 (6.78%)	0	1.53	0.216
	Basal ganglia	4	4 (6.78%)	0		
	Brainstem	32	21 (35.59%)	11 (44.0%)		
	Corpus callosum	8	7 (11.86%)	1 (4.0%)		
	Thalamus	31	20 (33.90%)	11 (44.0%)		
	Cerebellar vermis	5	3 (5.08%)	2 (8.0%)		
Operation						
	Biopsy	21	17 (28.81%)	4 (16.0%)	3.767	0.052
	Partial resection	31	23 (38.98%)	8 (32.0%)		
	Subtotal resection	23	15 (25.42%)	8 (32.0%)		
	Total resection	9	4 (6.78%)	5 (20.0%)		
Treatment						
	Radiotherapy	55	43 (72.88%)	12 (48.0%)	4.809	0.028
	Without Radiotherapy	29	16 (27.12%)	13 (52.0%)		
	Chemotherapy	38	30 (50.85%)	8 (32.0%)	2.172	0.141
	Chemotherapy + targeted therapy	11	8 (13.56%)	3 (12.0%)		
	Without chemotherapy	35	21 (35.59%)	14 (56.0%)		
OS^**^		84	14.48 ± 12.20	15.45 ± 13.89	0.32	0.75

* *World Health Organization*.

** *Overall survival*.

### Feature Selection and Model Comparison

The extracted texture parameters needed to be normalized and standardized to reduce bias due to the missing values and outliers. The best non-redundant features were then selected from the extracted features using the variance method and mRMR. Finally, five features were extracted each from T2w, T2 FLAIR, and T1c images, and 12 features were extracted for the combined model (Table 3).

**Table 3:** Best characteristic parameters of the four models.

**Dataset**	**Number**	**Characteristic parameters**
T2w	5	T2w_wavelet.HLL_first order_10 Percentile T2w _exponential_glszm_Gray Level NonUniformity T2w _gradient_glcm_MCC T2w _exponential_glcm_Inverse Variance T2w_wavelet.LHH_glszm_Low Gray Level Zone Emphasis
T2 FLAIR	5	T2 FLAIR_square_glszm_Small Area Low Gray Level Emphasis T2 FLAIR_wavelet.HLH_first order_Skewness T2 FLAIR_wavelet.LHH_gldm_Small Dependence High Gray Level Emphasis T2 FLAIR_wavelet.HHH_glszm_High Gray Level Zone Emphasis T2 FLAIR_wavelet.HHL_glcm_MCC
T1c	5	T1c_exponential_glszm_Small Area Low Gray Level Emphasis T1c _wavelet.HHH_glcm_Cluster Shade T1c _logarithm_glszm_Gray Level NonUniformity T1c _wavelet.LHH_gldm_Large Dependence High Gray Level Emphasis T1c _wavelet.LHH_glszm_Gray Level Variance
Combined model	12	T1c_exponential_glszm_Small Area Low Gray Level Emphasis T2 FLAIR_square_glszm_Low Gray Level Zone Emphasis T2 FLAIR_wavelet.HLH_first order_Skewness T2w_exponential_glszm_Gray Level NonUniformity T2 FLAIR_gradient_glszm_Small Area Low Gray Level Emphasis T1c_wavelet.HHH_glcm_ClusterShade T2 FLAIR_wavelet.HHH_glszm_High Gray Level Zone Emphasis T2w_wavelet.LHH_glszm_Low Gray Level Zone Emphasis T2w_wavelet.HLL_gldm_Dependence NonUniformity Normalized T1c_wavelet.HLH_glszm_High Gray Level Zone Emphasis T2w_gradient_glcm_MCC T2w_exponential_glcm_Inverse Variance

**Figure 4:**
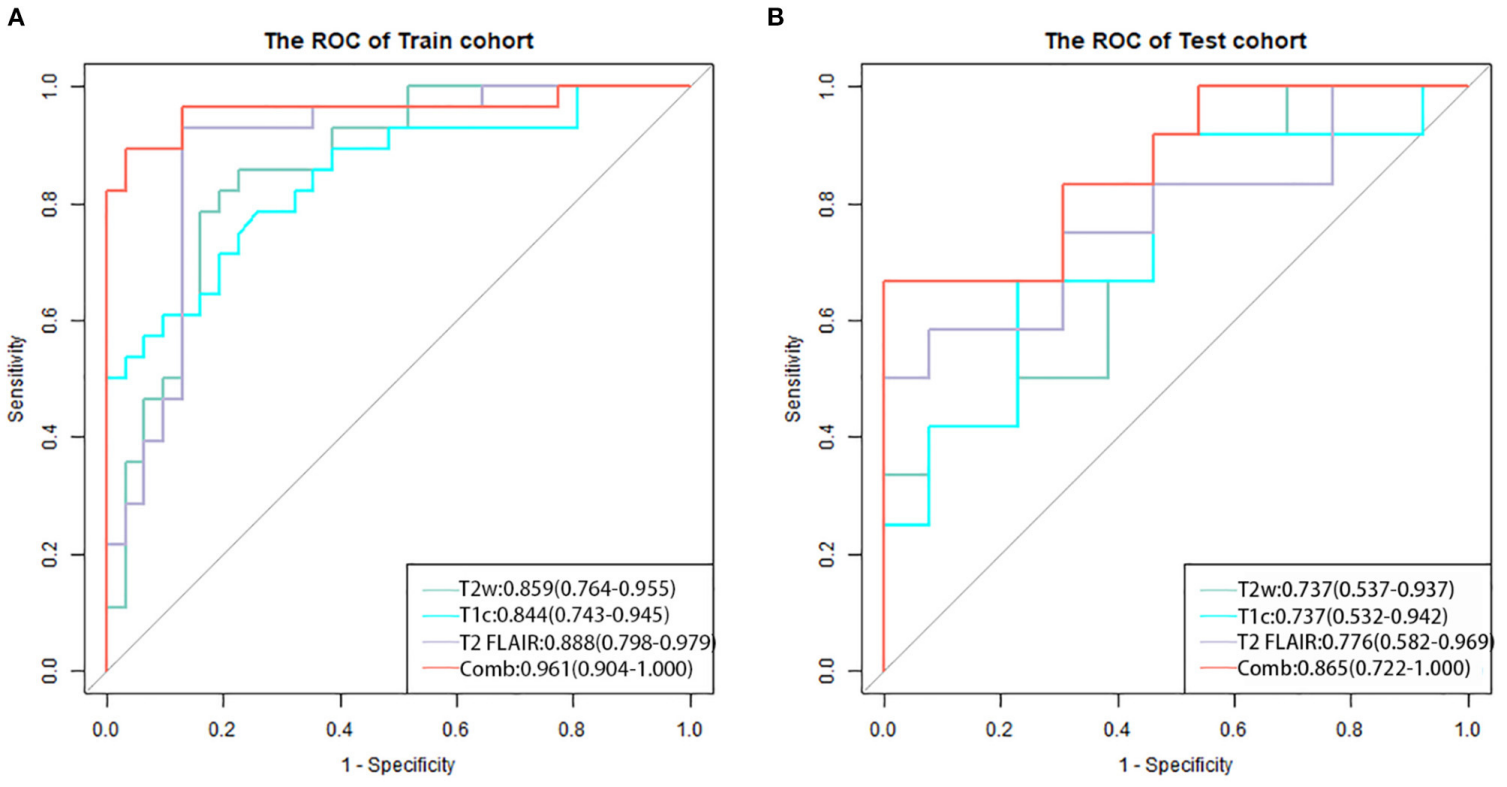
The receiver operating characteristic curves of the four models for the training **(A)** and test **(B)** cohorts. The combined model (red line) applied to the training and test cohorts had the best performance.

**Figure 5:**
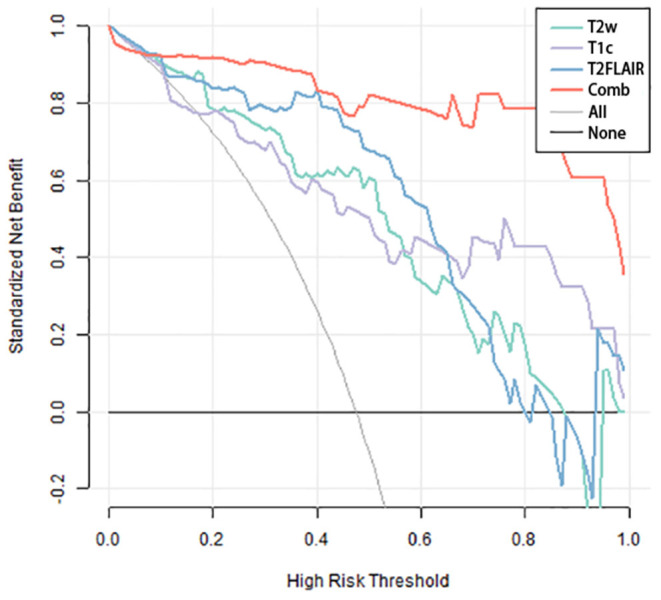
Decision-curve analysis of the four models. The x-axis represents the threshold probability, and the y-axis represents the net benefit. The combined model (red line) had a higher net benefit and better application value for predicting the survival time of patients with midline gliomas.

**Figure 6:**
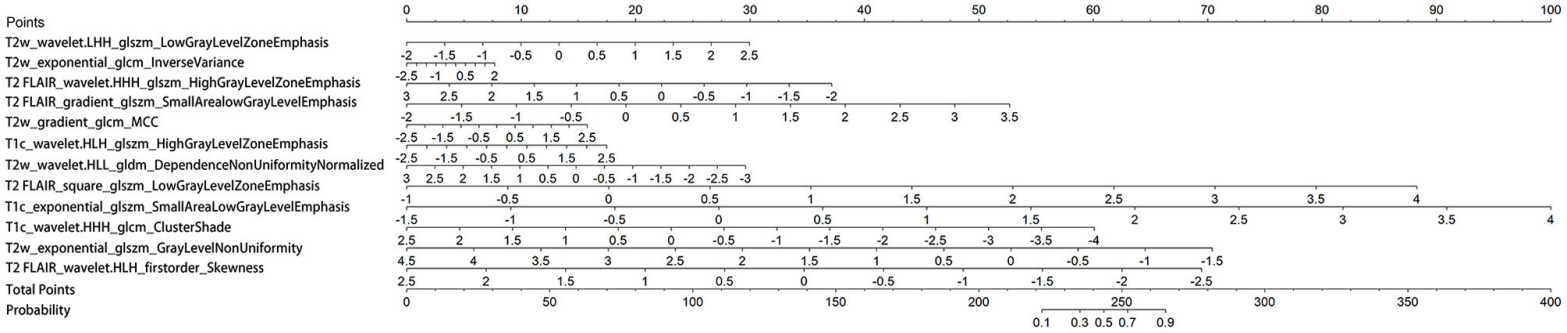
Nomogram of the combined model. A vertical line was drawn from each predictor to the point to obtain the score of the predictor. The scores of each predictor are then added, and a vertical line is drawn from the total score to the survival probability axis. The total score corresponds to the probability of >12 months survival.

The three separate models and the combined model performed well on both the training and test cohorts, shown by the high AC. The performance of each model on the training and test cohorts, respectively, were: T2w: AUC = 0.86 vs. 0.734, AC = 0.84 vs. 0.72; T2 FLAIR: AUC = 0.89 vs. 0.78, AC = 0.90 vs. 0.76; T1c: AUC = 0.84 vs. 0.74, AC = 0.76 vs. 0.72; combined model: AUC = 0.96 vs. 0.87, AC = 0.93 vs. 0.84 (Table 4). The combined model had the best performance. The ROC curves of the T2w, T2 FLAIR, T1c, and combined models are shown in Figure 4. DCA of the four models showed that the combined model offered the largest net benefit (Figure 5). The radiomics nomogram of the combined model applied to the training cohort showed that it directly predicted the probability of OS > 12 months in patients with midline gliomas (Figure 6).

**Table 4:** Comparison of the diagnostic efficiency between the four models.

**Dataset**	**AUC (95% CI)**		**Accuracy**	**Sensitivity**	**Specificity**
T2w	Training cohort	0.859 (0.764–0.955)	0.814	0.857	0.774
	Test cohort	0.737 (0.537–0.937)	0.72	0.917	0.538
T2 FLAIR	Training cohort	0.888 (0.798–0.979)	0.898	0.929	0.871
	Test cohort	0.776 (0.582–0.969)	0.76	0.583	0.923
T1c	Training cohort	0.844 (0.743–0.945)	0.763	0.786	0.742
	Test cohort	0.737 (0.532–0.942)	0.72	0.917	0.538
Combined model	Training cohort	0.961 (0.904–1.0)	0.932	0.893	0.968
	Test cohort	0.865 (0.722–1.0)	0.84	0.667	1.0

## Discussion

In this study, for the reproducibility and replicability of feature extraction, we used an open-source Pyradiomics 3.0.1 package of Python to calculate the features. To minimize the bias stemming from the use of data acquired at a single center and parameters of a single machine, we collected MRI data from different suppliers and different imaging protocols to recognize the diversity of data. Finally, we obtained optimal radiomics characteristics of T2w, T2 FLAIR, and T1c images to construct four models to non-invasively predict the prognosis of patients with midline gliomas. We compared the four models in terms of ROC curve, AUC, AC, sensitivity, and specificity and found that all models performed well on both the training and test cohorts, showing high AC. Among these, the combined model had the best performance. Moreover, DCA showed that the combined model offered the highest net benefit and application value. Our results demonstrate the feasibility of evaluating prognoses of patients with midline gliomas using traditional MRI radiomics. Furthermore, the fusion of radiomics characteristics obtained from different imaging sequences may improve the performance of the predictive model.

Numerous studies have shown that a single radiomics predictive model performs worse than a fusion radiomics model. Fusion radiomics models perform better and include more useful features, demonstrating the multimodal images play an important role in the prognostic evaluation of gliomas (23, 24). In this study, we constructed a machine learning model based on multi-parameter MRI data to predict the OS of patients with midline gliomas. Our results also showed that the fusion model performed better than the single radiomics model. In our training cohort, the combined model included 12 radiomics features (three features from T1c data, four features from T2 FLAIR data, and five features from T2w data), which were mainly derived from second- and higher-order texture features. The higher-order texture features can capture deeper imaging heterogeneity and provide information on tissue microstructure and local tumor microenvironment, which can help predict patient survival time (25, 26). Higher-order radiomics characteristics have also been shown to be closely related to genetic changes in tumors (6, 27–31). Five features in our combined model come from T2w, indicating that T2w images may provide more valuable texture features than T2 FLAIR and T1c images for evaluating survival time, which may explain why most studies prefer T2w sequences as the first choice for separate or joint analyses (26). However, T2 FLAIR and T1c images can offer additional details to the model and are thus indispensable (6, 27, 28). Therefore, we chose these three sequences. In addition, we applied the 12 features of the combined model to the nomogram, which enabled a more intuitive prediction of survival time.

To the best of our knowledge, the H3 K27M mutation, an independent prognostic factor, is common in midline gliomas. Several studies on the prediction of H3 K27M mutation status using machine learning based on radiomics features extracted from MRI data have been conducted to date. Su et al. (32) retrospectively studied 100 patients with midline gliomas and built 10 models using the Tree-based Pipeline Optimization Tool-based automated machine learning algorithm with radiomics features extracted from T2 FLAIR images. The AC of the best pipeline ranged from 0.788 to 0.867 in the training cohort and from 0.60 to 0.84 in the testing cohort. Pan et al. (14) retrospectively evaluated 151 brainstem gliomas and built a prediction model that incorporated 36 MRI features and three clinical features using a random forest algorithm. The model achieved an AC of 84.44% in the test cohort. Moreover, they constructed a simplified model that achieved an AC of 75.55% in the test cohort. Kandemirli et al. (33) developed an extreme gradient boosting algorithm classifier based on machine learning to predict the H3 K27M mutation using conventional MRI sequences. The model had an AUC of 0.791 in the training set and 0.737 in the test set, and the AC of the model was 73% in the test set. Furthermore, Li et al. (34) explored visually accessible MRI features of 30 patients with DMGs with and without the H3 K27M mutation using principal component analysis based on radiomics and found that T2w sequences may be more valuable and that cystic formation may be a biomarker for diagnosing DMGs with an H3 K27M mutation.

In addition to predicting the mutation status of H3 K27M, several researchers have evaluated the OS of patients with midline glioma using texture analysis. Szychot et al. (35) retrospectively analyzed 32 children with DMGs using a T2w imaging and apparent diffusion coefficient map-based texture analysis to predict outcomes of patients with DMG and found that the best predictor was mean of positive pixels, which could divide patients into poor and good prognosis groups according to a median survival time of 7.5 and 17.5 months, respectively. In this article, we included a larger number of cases to predict the OS of midline glioma patients using machine learning based on multiparametric MRI radiomics features, and our ACs were similar to those of the abovementioned machine learning studies. Thus, our model may offer an alternative method to predict the OS of patients with midline gliomas.

The current study has several limitations. First, a relatively small sample size and lack of an independent external test dataset in this study, which we will take as the key work in the next step. Second, the use of a single center may have introduced some bias. The next step is to conduct a multicenter study to improve the performance of the predictive model using more data. Third, only one method was used to build the model. If multiple methods were used to build and verify the models, a better model could be developed with improved AC. Forth, the patients who underwent radiotherapy in the long-term group are more than those in the short-term group, and some RT patients were treated with chemotherapy and/or targeted therapy at the same time, so it cannot be simply considered that RT can better predict the survival. In the next step, more cases need to be collected for subgroup analysis to detect the impact of RT on predicting the survival of DMG. Fifth, At present, there are many methods for extracting image features, such as local binary pattern (LBP), local ternary pattern (LTP), histogram of oriented gradients (HOG), etc. We used only one conventional method for feature extraction in this study due to the issues with overfitting and related techniques, In the future, we will try to use more methods to extract image features for further research.

Our radiomics model based on traditional MRI sequence data, especially the fusion radiomics model, may offer a reliable and non-invasive method to predict the survival time of patients with midline gliomas. Although there are several limitations, our findings provide a reference for the preoperative prediction and individualized treatment of gliomas as well as for further radiomics research on midline gliomas.

## Data Availability Statement

The original contributions presented in the study are included in the article/Supplementary Material, further inquiries can be directed to the corresponding authors.

## Ethics Statement

The studies involving human participants were reviewed and approved by IRB of Guangdong Sanjiu Brain Hospital. Written informed consent from the participants' legal guardian/next of kin was not required to participate in this study in accordance with the national legislation and the institutional requirements. Written informed consent was obtained from the individual(s), and minor(s)' legal guardian/next of kin, for the publication of any potentially identifiable images or data included in this article.

## Author Contributions

D-BD and QZ: conceptualization. D-BD: methodology, formal analysis, and writing—original draft preparation. Y-TL: software. W-SW and QZ: validation. J-FZ: investigation. PH: resources. S-NW, PH, and L-NC: data curation. W-SW: visualization. QZ: supervision and project administration. All authors: writing—review and editing, read, and agreed to the submission of the manuscript.

## Conflict of Interest

Y-TL was employed by GE Healthcare. The remaining authors declare that the research was conducted in the absence of any commercial or financial relationships that could be construed as a potential conflict ofinterest.

## Publisher's Note

All claims expressed in this article are solely those of the authors and do not necessarily represent those of their affiliated organizations, or those of the publisher, the editors and the reviewers. Any product that may be evaluated in this article, or claim that may be made by its manufacturer, is not guaranteed or endorsed by the publisher.
